# Institutional Needs

**Published:** 1916-01-29

**Authors:** 


### Institutional Needs.

TRI-MUR-TI PEPPERMINT CREAM.

(The Trimurti Company, Hills Place, Oxford Street, London, W.)

The use of liquid paraffin in the treatment of constipation has been steadily growing in recent years, and at the present time it is very largely prescribed by the medical profession, and still more largely employed as a popular medicine. The principle on which this form of treatment is based is utilised by the makers of Tri-Mur-Ti Cream, which is a very palatable preparation containing white Solidified Petroleum, Mitcham oil of peppermint and saccharin. The peppermint oil is added not merely as a flavouring agent but for its therapeutic action as a carminative and its antiseptic properties. The combination seems to be a good one, and patients to whom the idea of the internal administration of liquid paraffin is repulsive would have less objection to taking a pleasantly flavoured preparation like Tri-Mur-Ti.

